# A Combined Proteomic and Transcriptomic Signature Is Predictive of Response to Anti-PD-1 Treatment: A Retrospective Study in Metastatic Melanoma Patients

**DOI:** 10.3390/ijms25179345

**Published:** 2024-08-28

**Authors:** Domenico Mallardo, Mario Fordellone, Andrew White, Jakob Vowinckel, Michael Bailey, Francesca Sparano, Antonio Sorrentino, Mario Mallardo, Bianca Arianna Facchini, Rosaria De Filippi, Gerardo Ferrara, Vito Vanella, Kristina Beeler, Paolo Chiodini, Alessandra Cesano, Sarah Warren, Paolo A. Ascierto

**Affiliations:** 1 Department of Melanoma, Cancer Immunotherapy and Development Therapeutics, Istituto Nazionale Tumori—IRCCS Fondazione “G. Pascale”, 80131 Napoli, Italy; dome.mallardo@gmail.com (D.M.); franci.sparano@gmail.com (F.S.); a.n.sorrentino89@gmail.com (A.S.); mario.mallardo39@gmail.com (M.M.); arianna.facchini96@gmail.com (B.A.F.); vitovanella1@gmail.com (V.V.); 2 Mental and Physical Health and Preventive Medicine, Medical Statistics Unit, University of Campania “Luigi Vanvitelli”, 81100 Naples, Italy; mario.fordellone@unicampania.it (M.F.); paolo.chiodini@unicampania.it (P.C.); 3 NanoString Technologies, Seattle, WA 98109, USA; azariahw79@gmail.com (A.W.); mbailey@nanostring.com (M.B.); acesano@essapharma.com (A.C.); s.e.warren@gmail.com (S.W.); 4 Biognosys AG, 8952 Schlieren, Switzerland; jakob.vowinckel@biognosys.com (J.V.); kristina.beeler@biognosys.com (K.B.); 5 Department of Clinical Medicine and Surgery, Università degli Studi di Napoli Federico II, 80138 Naples, Italy; rdefilip@unina.it; 6 Department of Pathology and Cytopathology, Istituto Nazionale Tumori IRCCS Fondazione “G. Pascale”, 80131 Napoli, Italy; gerardo.ferrara@istitutotumori.na.it

**Keywords:** metastatic melanoma, immune checkpoint inhibitor, response biomarker, gene signature, protein signature

## Abstract

Resistance biomarkers are needed to identify patients with advanced melanoma obtaining a response to ICI treatment and developing resistance later. We searched a combination of molecular signatures of response to ICIs in patients with metastatic melanoma. In a retrospective study on patients with metastatic melanoma treated with an anti-PD-1 agent carried out at Istituto Nazionale Tumori—IRCCS—Fondazione “G. Pascale”, Naples, Italy. We integrated a whole proteome profiling of metastatic tissue with targeted transcriptomics. To assess the prognosis of patients according to groups of low and high risk, we used PFS and OS as outcomes. To identify the proteins and mRNAs gene signatures associated with the patient’s response groups, the discriminant analysis for sparse data performed via partial least squares procedure was performed. Tissue samples from 22 patients were analyzed. A combined protein and gene signature associated with poorer response to ICI immunotherapy in terms of PFS and OS was identified. The PFS and OS Kaplan–Meier curves were significantly better for patients with high expression of the protein signature compared to patients with low expression of the protein signature and who were high-risk (Protein: HR = 0.023, 95% CI: 0.003–0.213; *p* < 0.0001. Gene: HR = 0.053, 95% CI: 0.011–0.260; *p* < 0.0001). The Kaplan–Meier curves showed that patients with low-risk gene signatures had better PFS (HR = 0 0.221, 95% CI: 0.071–0.68; *p* = 0.007) and OS (HR = 0.186, 95% CI: 0.05–0.695; *p* = 0.005). The proteomic and transcriptomic combined analysis was significantly associated with the outcomes of the anti-PD-1 treatment with a better predictive value compared to a single signature. All the patients with low expression of protein and gene signatures had progression within 6 months of treatment (median PFS = 3 months, 95% CI: 2–3), with a significant difference vs. the low-risk group (median PFS = not reached; *p* < 0.0001), and significantly poorer survival (OS = 9 months, 95% CI: 5–9) compared to patients with high expression of protein and gene signatures (median OS = not reached; *p* < 0.0001). We propose a combined proteomic and transcriptomic signature, including genes involved in pro-tumorigenic pathways, thereby identifying patients with reduced probability of response to immunotherapy with ICIs for metastatic melanoma.

## 1. Introduction

The prognosis of patients with metastatic melanoma has been improved by the introduction of immune checkpoint inhibitors (ICI), which are now the standard of care. Treatment with ICIs provides a sustained response, still ongoing in more than 90% of patients with a follow-up of 7.9 months [1], with overall survival (OS) of over 50% at 5 years [2], and 57% at 6.5 years [3]. Indeed, immunotherapy targets and activates the memory of the host adaptive immune system, possibly explaining the prolonged activity. Nevertheless, many patients obtaining a response to ICI treatment develop resistance later [4]. Aiming at further improving outcomes, identifying resistance hallmarks may help to detect patients with low probability of benefit from immunotherapy and also to find new targets to overcome the mechanisms of resistance. Circulating biomarkers and tumor molecular signatures are widely investigated in advanced melanoma. They are associated with the response to ICIs, but reliable predictive markers for metastatic melanoma patients are still scant and have little reliabity [5,6]. Specifically, the only tissue markers for melanoma are tumor mutational burden (TMB) and microsatellite instability (MSI), based on results of KEYNOTE-158, but several criticisms have been raised in relation to this [7].

It has been found that the response to anti-PD-1 therapy in advanced melanoma is related to protein processing in the endoplasmic reticulum, and also to genes involved in the immune and inflammatory responses. The OS of patients with high expression of a two-protein based predictor was significantly better than the survival of patients with low expression (hazard ratio: 0.3189, 95% CI 0.0854–0.7382; *p* = 0.0125), suggesting that a combined proteomic and genetic analysis of tissue samples may be predictive of the response to immunotherapy in patients with advanced melanoma [8]. 

We decided to integrate a whole proteome profiling of metastatic tissue with targeted transcriptomics to search for a combination molecular signature of response to ICIs in patients with metastatic melanoma.

## 2. Results

**Figure 1:**
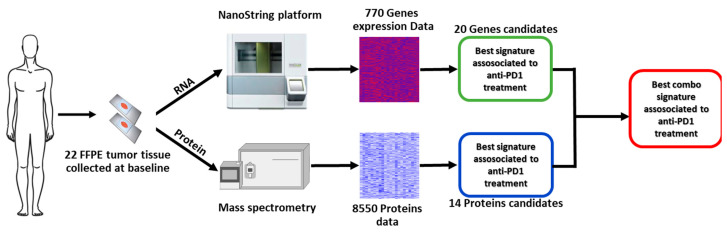
Study workflow.

Figure 1 shows the work flow of the study. Overall, tumor tissue samples from 22 patients (11 males and 11 females) were analyzed. The median age of patients was 66 years (IQR, 23.2), and *BRAF* mutation was detected in seven (31.8%) patients, central nervous system (CNS) metastases were present in five (22.7%) patients, and 16 (72.7%) subjects received nivolumab, while six (27.3%) received pembrolizumab (Table 1). Ten patients were classified as non-responders and 12 as responders. Table 1 shows the characteristics of the whole population and the two subgroups at the baseline. 

**Figure 2:**
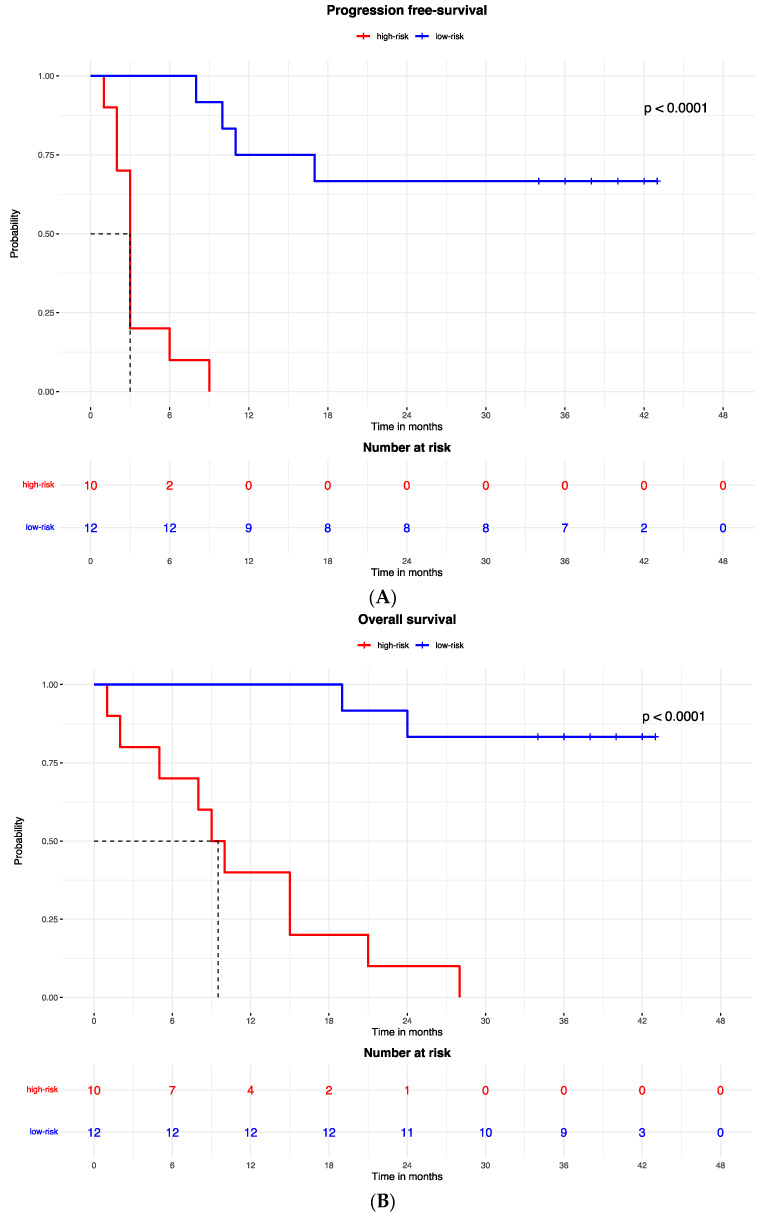

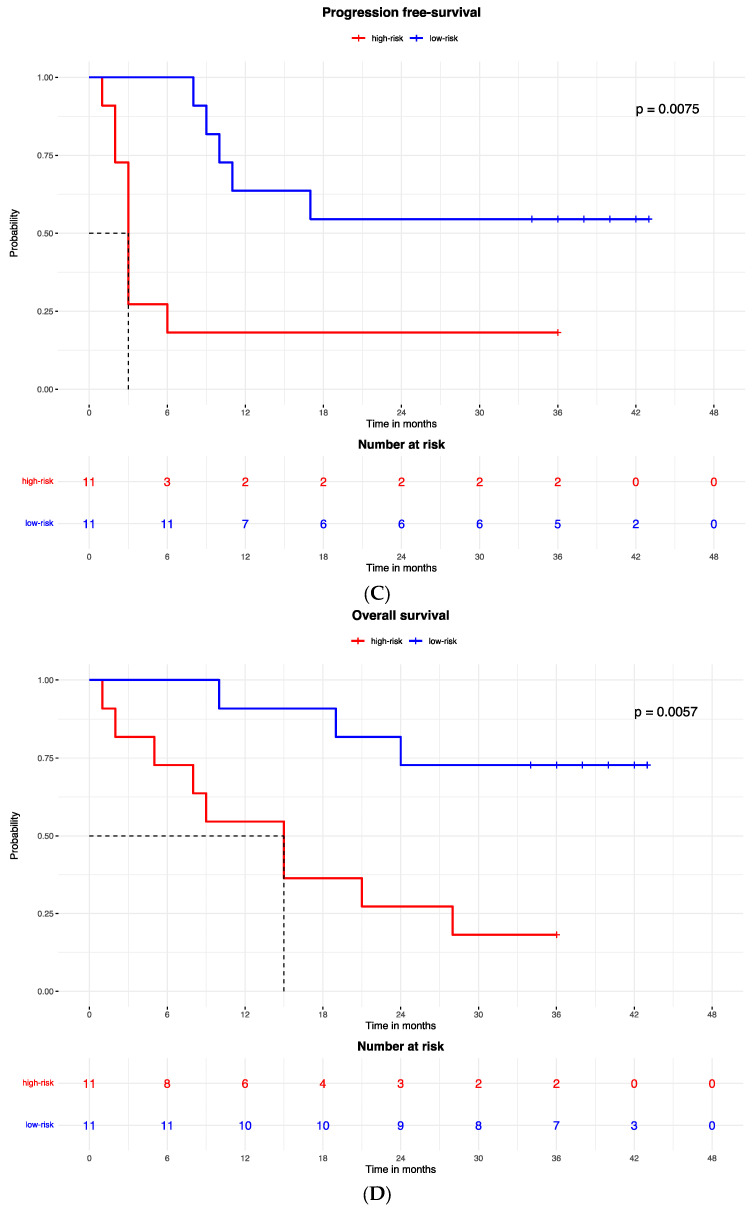

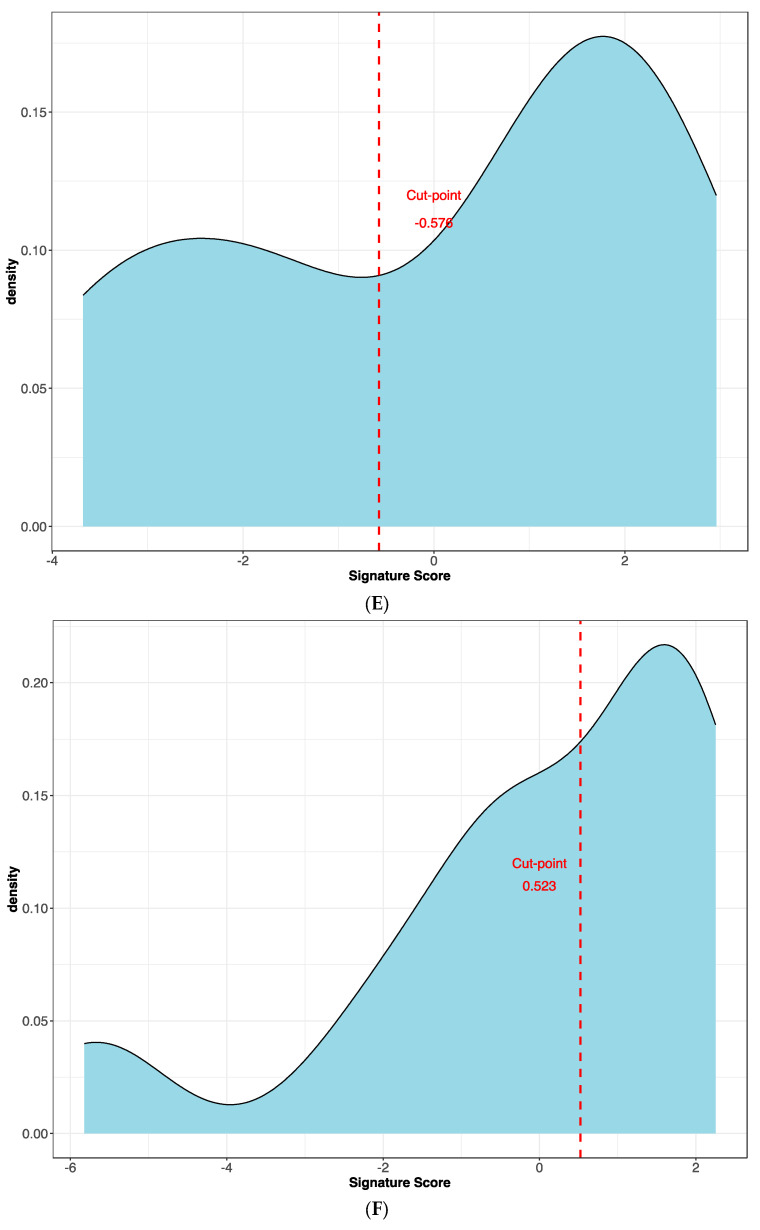
Kaplan–Meier curves for (**A**) the progression-free survival and (**B**) the overall survival of patients with high or low expression of the protein signature, (**C**) progression-free survival and (**D**) overall survival according to gene signature expression. Lasso regression for determination of best cut-point of protein (**E**) and gene signature (**F**).

**Figure 3:**
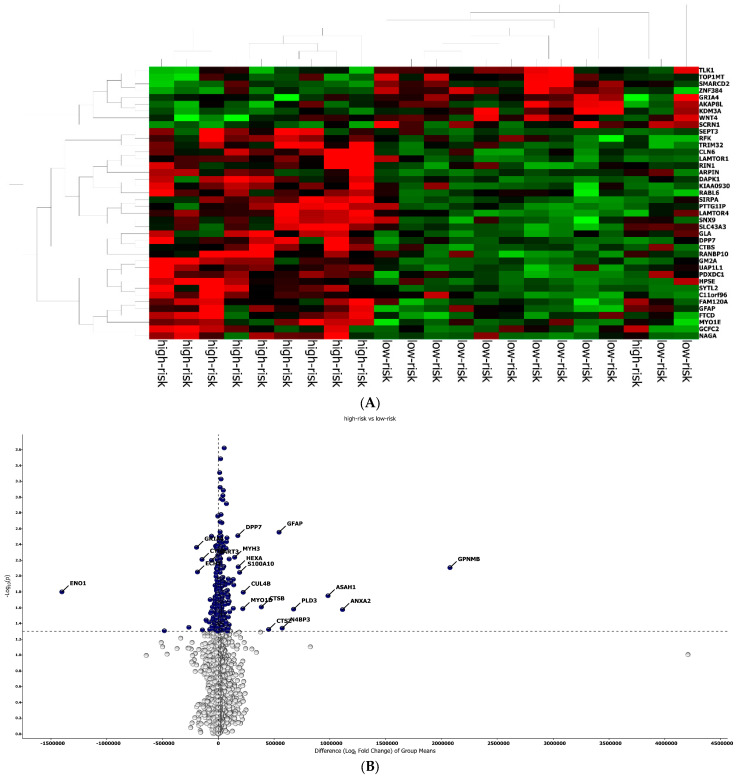
Protein analysis in high/low-risk patients. (**A**) Heat map representation. (**B**) Volcan plot showing gene distribution; *p*-values are reported on the *Y*-axis; values reported over the horizontal dotted line are significant, ±1 fold change is defined by vertical dotted.

The best protein signature associated with response to anti-PD-1 treatment included 14 proteins (*ZNF384*, *UAP1L1*, *TRIM32*, *TLK1*, *SYTL2*, *SIRPA*, *RANBP10*, *NAGA*, *MYOIE*, *LAMTOR4*, *GM2A*, *FAM120A*, *DAPK1*, *CLN6*) and discriminated genes associated with a high risk of disease progression (Supplementary Figure S1A). The optimal cut-off value (−0.578) of signature expression was selected through Lasso regression (Least Absolute Shrinkage and Selection Operator) (Figure 2E,F). The protein signature score was significantly higher in patients classified as responders (low-risk group) to the treatment than in non-responders (high-risk group) (*p* < 0.0001, Supplementary Figure S2A). Moreover, a different protein pattern was observed from the comparison of two subgroups, high risk vs. low risk (Figure 3).

Accordingly, the PFS Kaplan–Meier curve was significantly better for patients in the low-risk group (with high expression of the protein signature) (HR = 0.023, 95% CI: 0.003–0.213; *p* < 0.0001; Figure 2A). The median PFS of high-risk patients (with low expression of the protein signature) was 3 months (95% CI: 2–3), while the low-risk group median PFS was not reached.

A similar result was observed for the OS, which was better in patients in the low-risk (high expression of protein signature) group compared to patients with low expression of the protein signature and high-risk group (HR = 0.053, 95% CI: 0.011to 0.260; *p* < 0.0001; Figure 3B). The median OS was 9.5 months (95% CI: 5 to 9.5) in the high-risk (low protein signature expression) group and was not reached in the low-risk group.

**Figure 4:**
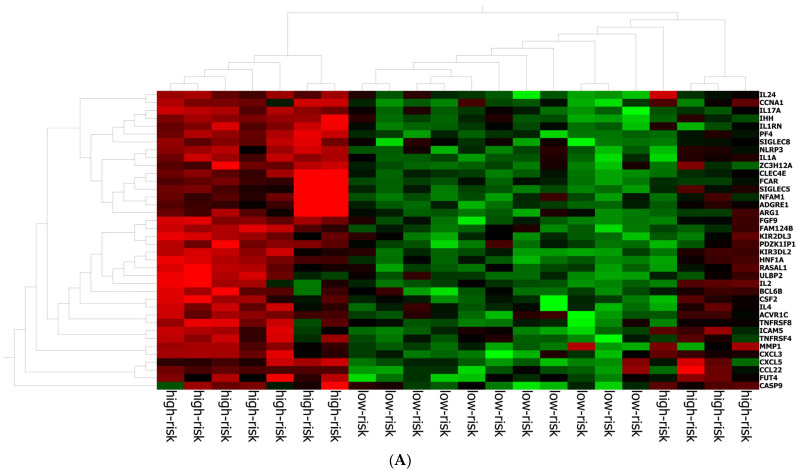

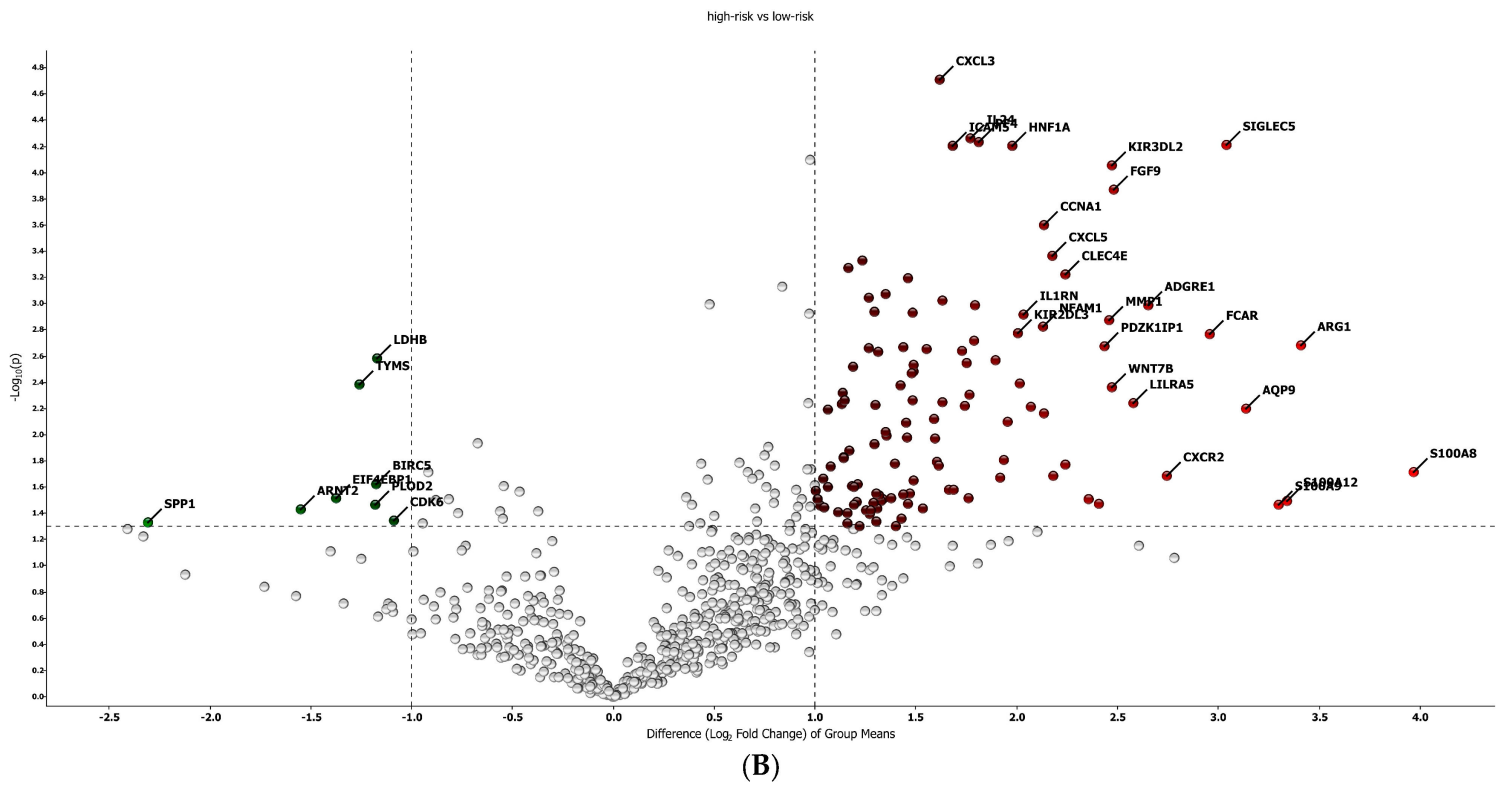
Gene expression in high/low-risk patients. (**A**) Heat map representation. (**B**) Volcan plot showing gene distribution; *p*-values are reported on the *Y*-axis; values reported over the horizontal dotted line are significant, ±1 fold change is defined by vertical dotted.

The best gene signature associated with response to anti-PD-1 treatment included 20 genes discriminating a pattern associated with a high risk (low expression of the gene signature) of progression (*VEGFA*, *SIFGLEC5*, *PF4*, *MAGEB2*, *LILRA5*, *ITGAM*, *IL24*, *IL2*, *IL1B*, *ICAM5*, *HNF1A*, *GOT1*, *FUT4*, *FGF9*, *FADD*, *CCNA1*, *CCL2*, *BIRC5*, *APH1B* and *ADGRE1*) (Figure 4A), and patients with high signature values had a high expression of genes with inflammatory and pro-tumoral activity, including *SIGLEC5*, *CXCL3*, *MMP1*, *ARG1*, *S100A8*, *S100A9* and *S100A12* (Supplementary Figure S3A). The optimal cut-off value (0.623) of signature expression was selected through Lasso regression (Figure 2F). Patients classified as non-responders (high risk) to anti-PD-1 treatment had a significantly lower gene signature expression (*p* < 0.0001; Supplementary Figure S2B) than responders (low risk). Moreover, a different genetic pattern was observed from the comparison of two subgroups, high risk vs. low risk (Figure 4).

The Kaplan–Meier curves showed that patients with low-risk gene signatures had better PFS (HR = 0 0.221, 95% CI: 0.071–0.68; *p* = 0.007; Figure 2C) and OS (HR = 0.186, 95% CI: 0.05–0.695; *p* = 0.005; Figure 2D). The median PFS was 3 months in the high-risk group and was not reached in the low-risk group. The median OS was 15 months in the high-risk group and was not reached in the low-risk group.

**Figure 5:**
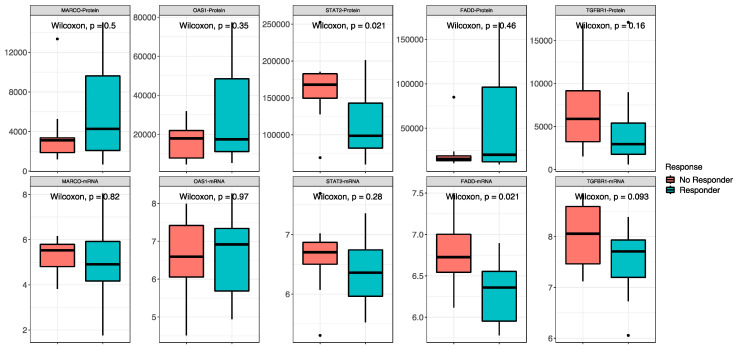
Combined proteomic and transcriptomic analysis. Non-responder/responder patients are defined by the red and blue boxes, respectively. On the top are the comparisons between proteins, and on the bottom are the comparisons between RNAs of the same molecules.

The proteomic and transcriptomic analyses were combined, and the top five candidate molecules between responders and non-responders were found among the 418 overlapping sets of genes and proteins (Figure 5). No difference was found between high- and low-risk patients for the expression of both protein and RNA of MARCO, OAS1 and TGFBR1. For STAT2, signal transducer and activator of transcription 2, a gene involved in cell replication and growth, the protein was less expressed in responders than in non-responders (low-risk vs. in high-risk patients) (*p* = 0.02), while there was no difference in RNA expression between the groups. FADD, Fas-associated via death domain, had a higher expression of RNA in non-responders (*p* = 0.02) and no difference in the protein expression. 

**Figure 6:**
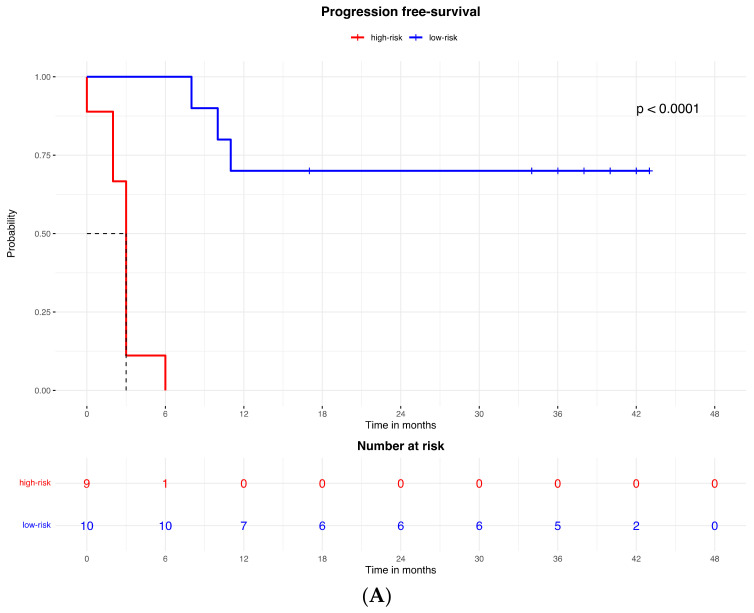

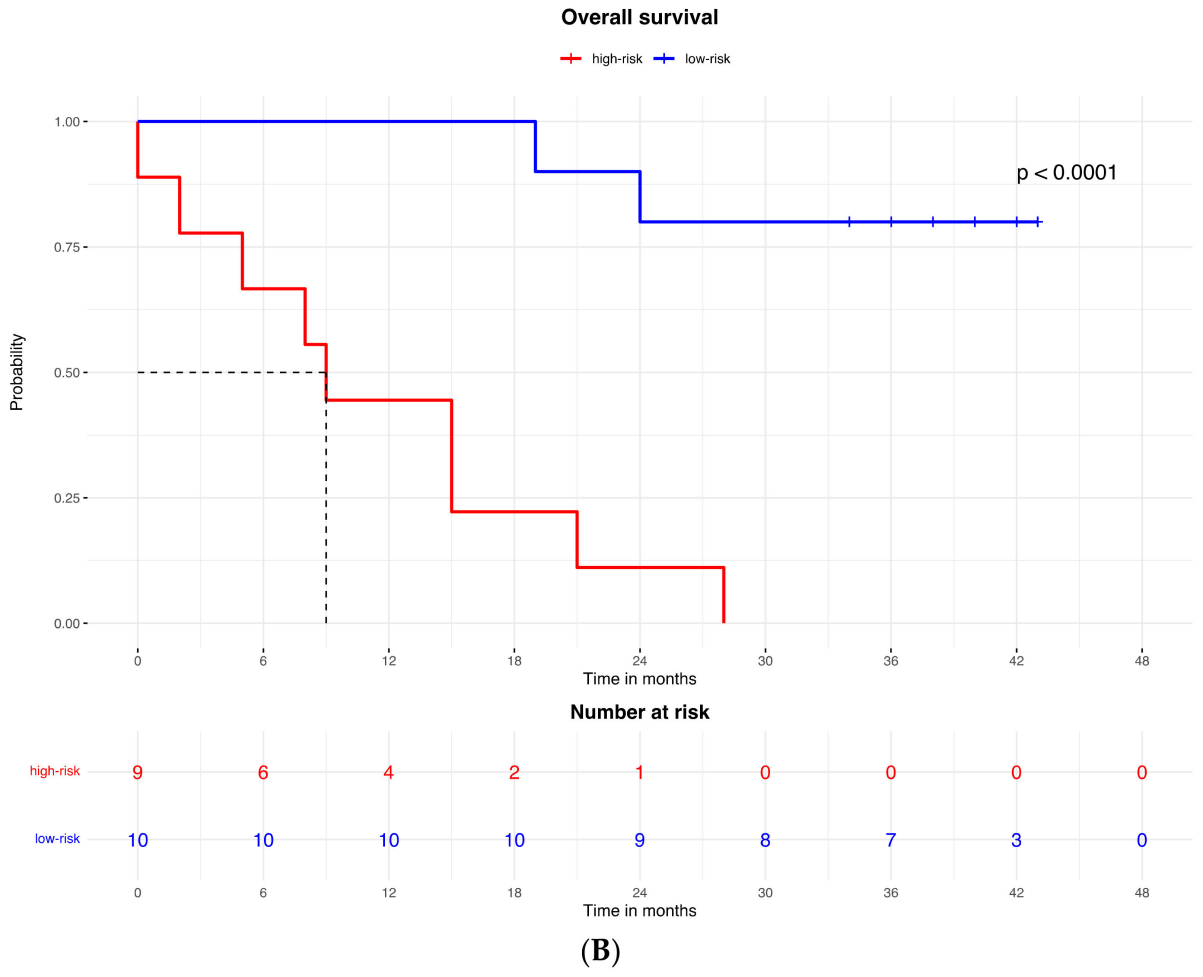
Kaplan–Meier curves for (**A**) progression-free survival and (**B**) overall survival according to the combined proteomic and transcriptomic signature expression.

The proteomic and transcriptomic combined analysis was significantly associated with the outcomes of the anti-PD-1 treatment. All the patients with low expression of protein and gene signatures had progression within 6 months of treatment (median PFS = 3 months, 95% CI: 2–3), with a significant difference vs. the low-risk group (median PFS = not reached; *p* < 0.0001; Figure 6A), and significantly poorer survival (OS = 9 months, 95% CI: 5–9) compared to patients with high expression of protein and gene signatures (median OS = not reached; *p* < 0.0001; Figure 6B). 

## 3. Materials and Methods

### 3.1. Patients

A retrospective study was carried out at Istituto Nazionale Tumori—IRCCS—Fondazione “G. Pascale”, Naples, Italy. The study was approved by the Ethics Committee of Istituto Nazionale Tumori—IRCCS—Fondazione “G. Pascale”, Naples, Italy, protocol number 32/22 oss. The study was performed in accordance with the revised version of the Declaration of Helsinki (52nd WMA General Assembly, Edinburgh, Scotland, October 2000).

Consecutive adult patients with histologically confirmed metastatic melanoma (stage IV, M category 1A–C, according to the American Joint Committee on Cancer AJCC 7th Edition) treated with an anti-PD-1 agent (either nivolumab or pembrolizumab) in the first line setting between August 2017 and March 2018, were included in the analysis. All patients provided their written informed consent to treatment and publication of anonymous data. 

### 3.2. Methods

#### 3.2.1. Survival Outcomes Measures 

RECIST 1.1 criteria were used to radiologically evaluate the tumor response as complete response (CR), partial response (PR), stable disease (SD) or progressive disease (PD). According to clinical benefit, patients were considered responders if they obtained CR, PR or SD > 1 year after anti-PD-1 treatment or non-responders if they had SD ≤ 1 year or PD. The following parameters were recorded and considered in the analysis for this study: progression-free survival (PFS)—calculated from the time of the first dose of anti-PD-1 agent to radiological progression, death or lost–to–follow–up, whichever occurred first; OS calculated from the time of the first dose of anti-PD1 agent to death or lost–to–follow–up, whichever occurred first.

#### 3.2.2. Transcriptomic and Proteomic Analysis

Metastatic tumor tissue samples were collected at the baseline, before the initiation of anti-PD-1 therapy, formalin-fixed and embedded in paraffin (FFPE). Consecutive tissue sample sections were used for proteomic and transcriptomic analyses; by this method, similar cells were present in the sections used for the two analyses.

Whole proteome profiling was obtained with Biognosys’ TrueDiscovery™ platform using advanced data-independent acquisition liquid chromatography–mass spectrometry (LC-MS) workflow adapted from Bruderer et al. [9]. A deep spectral library was generated, and proteins were quantified using the Spectronaut™ v17 software (Biognosys AG, Schlieren, Switzerland).

RNA was extracted from the same tumor samples using QIAmp RNA FFPE Tissue Kit (Qiagen Sciences, Germantown, MD, USA). Purified RNA was used for hybridization and underwent gene profiling analysis on NanoString nCounter through the IO 360 panel (PanCancer IO 360™ Panel, NanoString, Seattle, WA, USA), characterized by human genes associated with immune activation, inflammation and control of the cell cycle. Gene data were normalized using nSolver Version 4.0 Software; NanoString. Counts were normalized to External RNA Controls Consortium technical controls and 30 housekeeping genes. Raw data generated in this study have been deposited in Zenodo: https://doi.org/10.5281/zenodo.11636595, accessed on 1 June 2024.

#### 3.2.3. Statistical Analysis 

Continuous variables were reported as either the means and standard deviation or median and interquartile ranges (IQRs) according to their distribution, as assessed by the Shapiro–Wilk normality test. Categorical variables were reported as percentages. Differences in characteristics of patients between the groups of responder and non-responder patients were tested by *t*-test or Wilcoxon test (according to their distribution) and Pearson’s chi–squared test or Fisher’s exact test for continuous and categorical variables, respectively. To measure the linear association between continuous variables, the Pearson correlation coefficient was used if variables had a normal distribution, otherwise Spearman’s correlation coefficient was calculated.

To identify the proteins and mRNA gene signatures associated with the patient’s response groups, the discriminant analysis for sparse data performed via partial least squares procedure was performed. The sparse variant of Partial Least Squares Discriminant Analysis (PLS-DA) enables the selection of the most predictive or discriminative features in the data to classify the samples [10].

The identified signature scores were used as synthetic risk indices, and to define the subgroups of low and high risk, a Lasso regression (Least Absolute Shrinkage and Selection Operator) was conducted through cross-validation to determine the optimal cut-point, minimizing the risk of overfitting. 

To assess the prognosis of patients according to groups of low and high risk, we used PFS and OS as outcomes. The differences in prognosis between the low-risk group and high-risk group were tested by log–rank test and represented by Kaplan–Meier curves. Median follow-up was estimated by the inverse Kaplan–Meier approach. Statistical tests with *p*-values < 0.05 were considered statistically significant. All the statistical analyses were performed with the R Studio Statistical software, version 4.1.3.

## 4. Discussion

Our group has widely investigated possible response markers that could guide therapeutic choice in patients with advanced melanoma [11,12,13]. The experience in the methods used in this area of research was applied to this retrospective study that identified a combined protein and gene signature associated with better response to ICI immunotherapy of patients with metastatic melanoma in terms of PFS and OS. The signature discriminates a gene pattern associated with the risk of progression and is related to the expression of genes involved in inflammation and tumor-promoting pathways. So, patients with a high signature expression have higher expression of pro-tumorigenic genes but have a better prognosis than non-responders after immunotherapy. Indeed, checkpoint inhibition seems to have a better efficacy when inflammation and tumor-promoting pathways are excessively represented. Indeed, proteins in the signature identified by Trilla-Fuertes et al. [8] are related to the immune status of several tumors.

Data from the OpACIN-neo trial have previously identified a gene IFN-γ signature predictive for favorable pathologic response and better outcomes in patients with stage III melanoma treated with ICIs [6,14]. In this case, the signature discriminates a subpopulation of patients with pre-activated immunity and better outcomes. The authors demonstrated that a combination of the signature with TMB increased predictivity. A Tumor inflammation signature (TIS) was identified for clinical response in 58 metastatic cancer patients treated with anti-PD-1, and the TIS score was characterized from the expression of 16 genes that were significantly associated with a response to anti-PD-1 treatment (OR = 2.64, 95% CI [1.4–6.0], *p* = 0.008) and with overall survival (HR = 0.37, 95% CI [0.18–0.76], *p* = 0.005 [15]. In another retrospective study there was evaluation of a protein signature from 69 melanoma lymph node metastases, in which seven proteins were significantly associated with patient survival [16]. Our study combined two different signatures and obtained a good correlation with prognosis in response to immunotherapy, with better predictivity than with RNA evaluation alone. The IFN-γ signature is composed of 10 genes (*IFN-γ*, *CCR5*, *CXCL11*, *IDO1*, *PRF1*, *GZMA*, *HLA-DRA*, *CXCL10*, *CXCL9* and *STAT1*) that play a role in the interplay of tumor cells with the tumor microenvironment and local immune response. It selects patients with a preactivated immune system [14].

The signature presented in our article includes proteins and RNAs related to the main tumor-promoting molecular pathways, and we suggest that it selects patients with a high expression of tumor-promoting mechanisms targeted by ICIs. Indeed, the Kaplan–Meier curves for PFS and OS of our patients receiving immunotherapy showed a significant difference between the group with low protein or gene signature, which had a poorer prognosis, and that with high protein or gene signature expression, respectively. Additionally, we observed a very poor prognosis in all patients with high expression of both signatures.

Combining protein and RNA signature evaluation, we observed that the STAT2 protein was less expressed in responder patients than in non-responders, while STAT2 RNA was not significantly different in responders than in non-responders. Additionally, although FADD RNA was more expressed in non-responders, protein expression was not different in the two groups of response. We can speculate that post-translational mechanisms may impact the maturation of RNA into protein in the metastatic tissue of responder patients, resulting in the modulation of tumor resistance pathways involving FADD and STAT2 [15,17]. FADD is a protein that interacts with the death domain of Fas and initiates apoptosis [18]. Fas, FADD and caspase 8 form a death-inducing signaling complex, triggering apoptosis through caspase 8 activation, which initiates the caspase cascade [10,11,12,13,14,15,16,17,18,19,20,21]. STAT2 is a mediator in the signaling pathway of type I interferons (IFN-α and IFN-β) that is involved in immune reactions regulating tumor cells’ capability to escape, survive and progress [22]. Following type I IFN binding to cell surface receptors, Jak kinases (TYK2 and JAK1) are activated, leading to tyrosine phosphorylation of STAT1 and STAT2. The phosphorylated STATs dimerize and associate with IRF9/ISGF3G to form a complex termed ISGF3 transcription factor that enters the nucleus. ISGF3 binds to the IFN-stimulated response element (ISRE) to activate the transcription of interferon-stimulated genes, which drives the cell to an antiviral state [23]. The monocentric retrospective nature and sample size are limitations of this study.

In conclusion, we propose a combined proteomic and transcriptomic signature, including genes involved in pro-tumorigenic pathways, that identifies patients with reduced probability of response to immunotherapy with ICIs for metastatic melanoma.

## Figures and Tables

**Table 1:** Characteristics of patients at baseline.

Characteristic	Response, n (%)		Overall (*n* = 22), *n* (%)
	Non-Responder (*n* = 10) ^†^	Responder (*n* = 12)	
Age	66.00 (16.75)	63.00 (28.50) ^†^	66.00 (23.25) ^†^
Gender			
Female	7.0 (70.0%)	4.0 (33.3%)	11.0 (50.0%)
Male	3.0 (30.0%)	8.0 (66.7%)	11.0 (50.0%)
*BRAF*			
Mutate	2.0 (20.0%)	5.0 (41.7%)	7.0 (31.8%)
Wild-Type	8.0 (80.0%)	7.0 (58.3%)	15.0 (68.2%)
LDH class			
135–225	3.0 (30.0%)	9.0 (75.0%)	12.0 (54.5%)
240–480	7.0 (70.0%)	3.0 (25.0%)	10.0 (45.5%)
M-Category			
M0	0.0 (0.0%)	0.0 (0.0%)	0.0 (0.0%)
M1A	2.0 (20.0%)	4.0 (33.3%)	6.0 (27.3%)
M1B	1.0 (10.0%)	2.0 (16.7%)	3.0 (13.6%)
M1C	7.0 (70.0%)	6.0 (50.0%)	13.0 (59.1%)
CNS			
No	5.0 (50.0%)	12.0 (100.0%)	17.0 (77.3%)
Yes	5.0 (50.0%)	0.0 (0.0%)	5.0 (22.7%)
Treatment			
Nivolumab	9.0 (90.0%)	7.0 (58.3%)	16.0 (72.7%)
Pembrolizumab	1.0 (10.0%)	5.0 (41.7%)	6.0 (27.3%)

^†^ Median (IQR). For each tissue sample, 8550 proteins and 770 RNAs were evaluated, with an overlap of 418 genes for which proteomic and transcriptomic data were profiled.

## Data Availability

The data presented in this study are openly available in Zenodo, at https://doi.org/10.5281/zenodo.11636595 accessed on 1 June 2024.

## Supplementary Materials

The following supporting information can be downloaded at: https://www.mdpi.com/article/10.3390/ijms25179345/s1.

## Abbreviations

CR	complete response
FFPE	formalin-fixed and embedded in paraffin
ICI	immune checkpoint inhibitor
IQR	interquartile ranges
MSI	microsatellite instability
OS	Overall survival
PD	progressive disease
PFS	progression-free survival
PR	partial response
ROC	receiver operating characteristic
SD	stable disease
TMB	tumor mutational burden
