# Supplementary material for: A Combined Proteomic and Transcriptomic Signature Is Predictive of Response to Anti-PD-1 Treatment: A Retrospective Study in Metastatic Melanoma Patients
Source: Int J Mol Sci. 2024 Aug 28;25(17):9345. doi: 10.3390/ijms25179345 (PMC11395026; doi:10.3390/ijms25179345)

Supplementary Figure S1. Protein signature identification (A) and selection of the cut-off value (B)

A

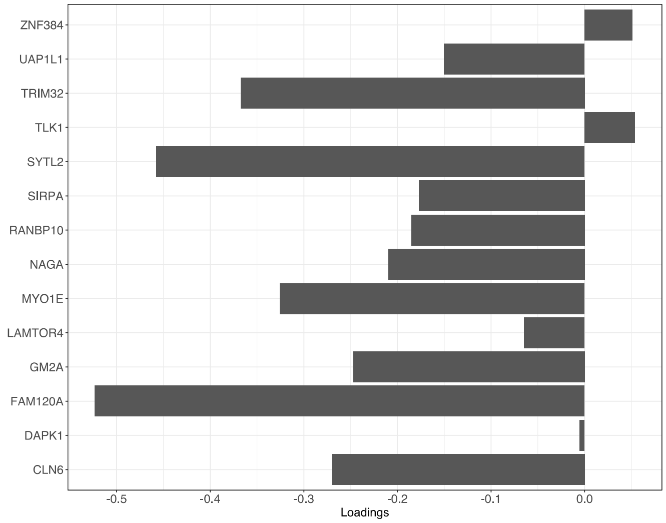

B

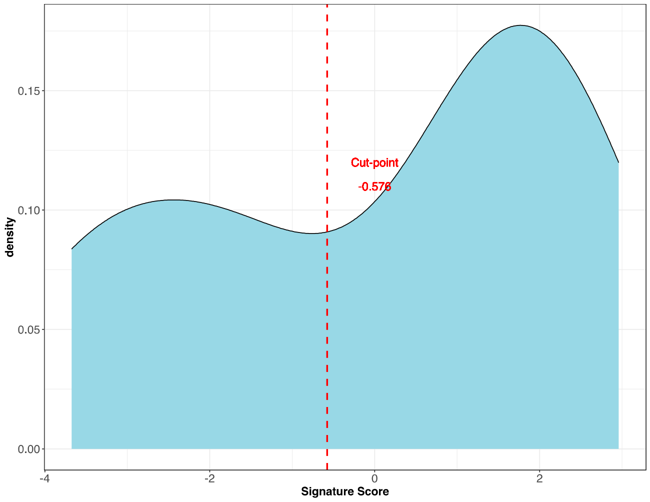

Supplementary Figure S2. Association of latent signature scores with response to anti-PD-1 treatment. (A) Protein signature. (B) Gene signature

A

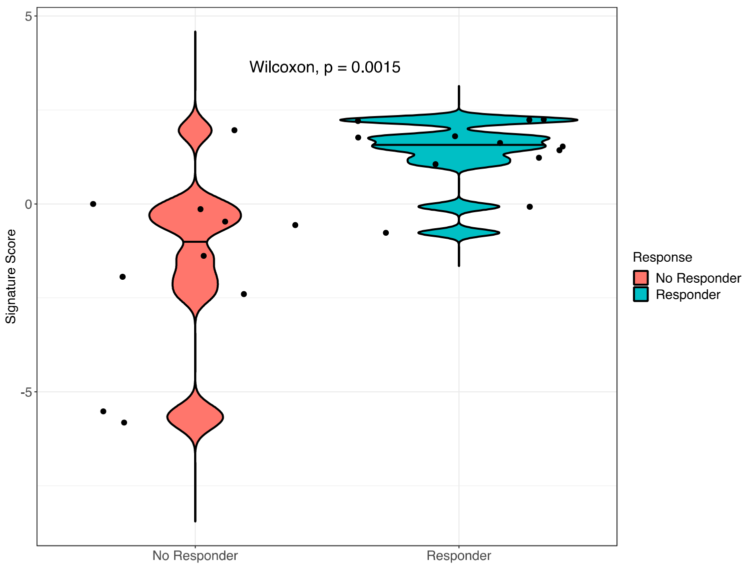

B

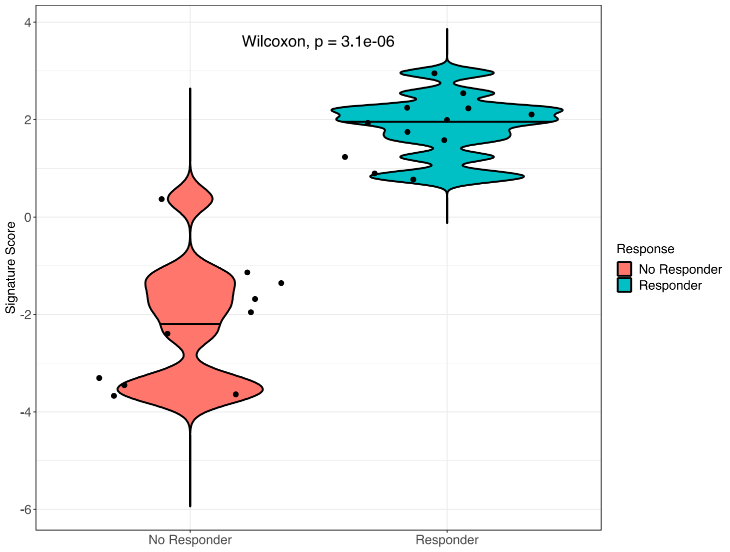

Supplementary Figure S3. A) mRNA signature identification and B) selection of the cut-off value (B)

A

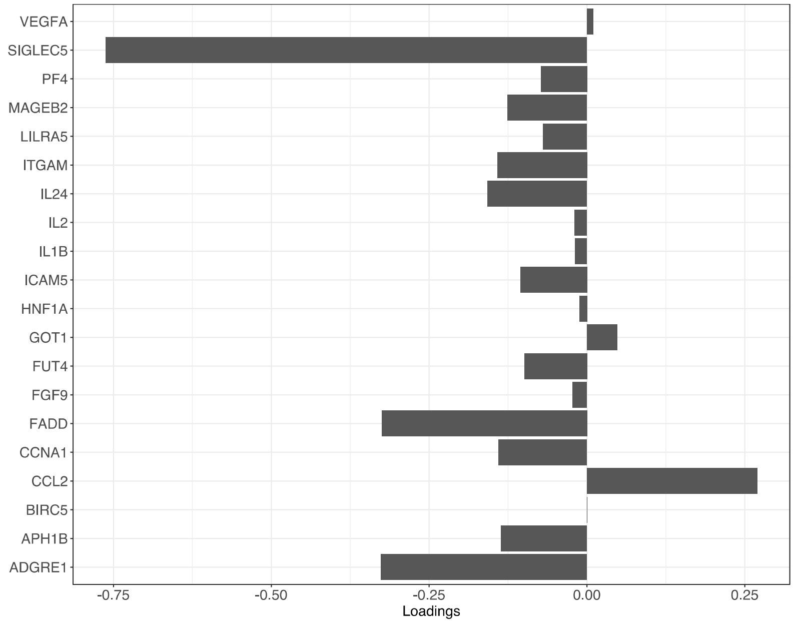

B

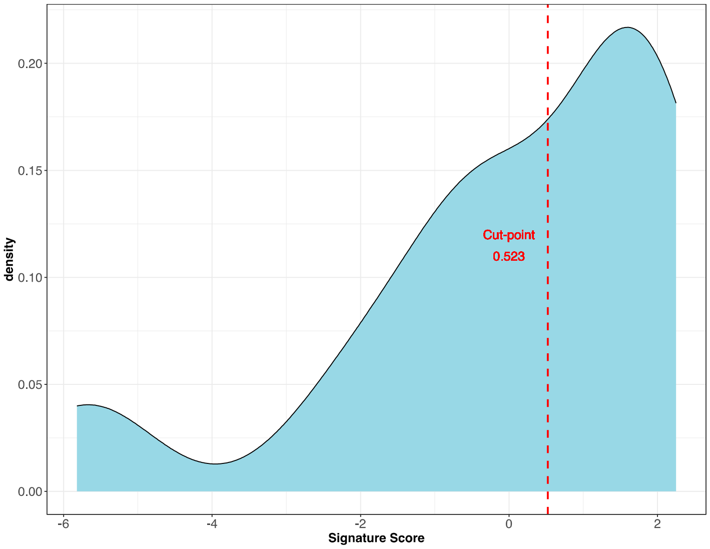

Supplement: Supplementary file 1 [file ijms-25-09345-s001.zip › ijms-3145569-supplementary.pdf]
